# Microfluidic contact lens: fabrication approaches and applications

**DOI:** 10.1038/s41378-025-00909-3

**Published:** 2025-04-03

**Authors:** Aravind M, Ankur Saxena, Dhaneshwar Mishra, Kulwant Singh, Sajan D. George

**Affiliations:** 1 Manipal Institute of Applied Physics, Manipal Academy of Higher Education, Manipal, 576104 India; 2 Department of Mechanical Engineering, Manipal University Jaipur, Jaipur, 303007 India; 3 Skill Faculty of Engineering & Technology, Shri Vishwakarma Skill University, Palwal, 121102 India; 4 Centre for Applied Nanosciences (CAN), Manipal Academy of Higher Education, Manipal, 576104 India

**Keywords:** Micro-optics, Nanoparticles

## Abstract

Microfluidic contact lenses integrate microscale features that can efficiently and precisely manipulate, interact, and analyze the small volumes of tears available in the limited accessible space for the lens in the eye. The microfluidic network on contact lenses allows the miniaturization of biochemical operations on the wealth of physiological information available in the eye. Sensors integrated into channels enable real-time monitoring of ocular parameters, including glucose, pH, electrolytes, or other biomarkers. Additionally, microchannel-integrated contact lenses have demonstrated potential as power-free, continuous intraocular pressure monitoring platforms for the effective management of glaucoma. Furthermore, the controlled release of medications directly onto the eye from microfluidic contact lenses enhances therapeutic efficacy by increasing bioavailability. Despite current challenges such as scalable fabrication techniques, microfluidic contact lenses hold immense promise for ocular health, bridging the gap between diagnostics and treatment. This review summarizes the progress made in the design and fabrication of microfluidic contact lenses, with a special emphasis on the methods adopted to fabricate microfluidic contact lenses. Furthermore, the various applications of microfluidic contact lenses, ocular disease diagnosis, and drug delivery in particular are discussed in detail. Aside from outlining the state-of-the-art research activities in this area, challenges and future directions are discussed here.

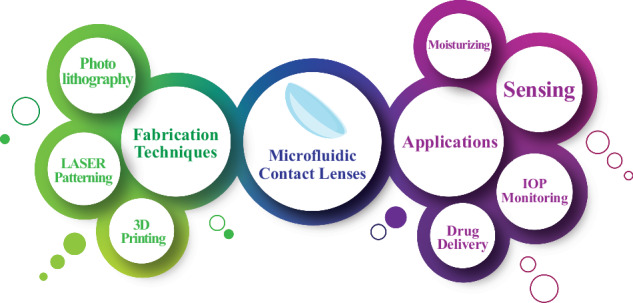

## Introduction

This decade is witnessing intense research to tailor contact lens-based platforms from mere vision correction devices to wearable smart devices that allow diagnosis and onsite treatment. The evolution from the first conceptualization by *Leonardo Da Vinci* and the manufacturing of the first powered contact lens by *Karl Otto Himmler* and *August Muller* to an augmented reality display on contact lenses is an outcome of advances in interdisciplinary research^1,2^. The refinement of contact lenses has been made possible by developments in the fields of material science, ophthalmology, and engineering, which have resulted in the transition from heavy-blown glass to daily disposable and extended-wear contact lenses with superior performance and comfort^3^. Contact lenses are primarily used for vision correction. Hence, leveraging this platform for ocular parameter sensing and drug delivery for treatment provides an added advantage. The cornea’s surface uniquely provides a convenient, minimally invasive interface to the body’s physiological conditions, making contact lenses a useful platform for disease diagnosis and drug delivery. The primary function of the contact lens is to rectify refractive errors such as myopia, farsightedness, and astigmatism^4,5^. However, recent advances illustrate that contact lenses can be employed to improve color perception in colorblind patients^6,7^. The contact lens is constant with the tear film, which is composed of the outermost lipid, middle aqueous, and inner mucin layers. A uniform lipid layer can inhibit the evaporation of the underlying aqueous layer^8^. Mucins aid in stabilizing and spreading the tear film and protect against desiccation and microbial invasion^9^. The middle aqueous layer contains electrolytes and proteins, and the thicker layer lubricates the surface of the eye^10^. There is a close correlation between certain metabolites in blood and tears due to plasma leakage, where certain components of blood pass through the blood‒tear barrier and enter the tear fluid^11,12^. This makes the tear fluid a surrogate for the analysis of blood composition for continuous and minimally invasive diagnosis. Additionally, the eye offers wider diagnostic potential as a sensing site; therefore, contact lens sensors have the potential to improve diagnosis and treatment. Widely studied diagnostic indicators using contact lens sensors include glucose monitoring among diabetes patients^13,14^, and keeping track of intraocular pressure (IOP) for glaucoma management^15,16^. The diagnostic capability is limited not only to IOP and glucose monitoring but also to monitoring lactate^17^, pH^18^, electrolytes^19^, protein^20^, moisture^21^, eye movement^22^, etc. The sensitive and selective detection of different ocular biomarkers is achieved by integrating different sensing techniques, such as spectroscopic^23,24^ electrical^15,25^, and colorimetric techniques^18^. The identification of biomarkers plays a crucial role in the diagnosis of various ocular diseases, often leading to subsequent treatment, and an ideal wearable platform should include the ability to administer medications. Drug delivery through contact lenses has emerged as an innovative approach for treating ocular diseases^26,27^. Compared with common eye drops, this approach offers advantages such as increased bioavailability, improved patient compliance, and a reduced risk of side effects^28^. Recent progress in the development of drug-delivery contact lenses has led to several therapeutic benefits, which can potentially revolutionize the treatment of ocular conditions such as glaucoma^29^, dry eye^30^, and corneal inflammation^31^. Research and developments on contact lenses have scopes beyond sensing and drug delivery, and complications associated with continuous contact lens wear, such as bacterial adhesion and protein deposition, are now addressed through surface-modified contact lenses^32,33^ and advancements in smart contact lenses, such as the integration of glucose fuel cells and supercapacitors^34,35^.

Several of the applications of contact lenses rely upon the ability to manipulate tear fluid precisely and incorporate various chemicals into the lenses. This becomes challenging owing to several inherent characteristics of contact lenses and the eye, especially the minimum volume of tear available and the limited space accessible on the lens. Microfluidic devices offer miniature platforms that can manipulate and analyze small volumes of fluids efficiently and precisely. These devices feature a network of microchannels combined to perform various functions, such as mixing, pumping, and containing. Microfluidic devices have been widely used for various biomedical applications because of their several advantages, such as low sample consumption, biocompatibility, transparency, and multifunction integration^36^. Microfluidic contact lenses (MCLs) have thus attracted particular interest in the last few years because of their ability to allow on-eye, real-time tear fluid processing. The crucial advantages lie in the reduced contamination, fast response time, continuous monitoring, integration with different sensing techniques, precision, and controlled release. The widely used application of MCLs is as a sensing platform and drug delivery device. The ability to direct tear flow and mix with different reagents allows MCLs to become efficient sensors. The deformation of the microfluidic network inside the soft MCL under fluctuating IOP and the displacement of fluid inside the microchannel is used for monitoring IOP variation. The microfluidic network to contain and release drugs without compromising the essential properties of contact lenses presents a promising alternative to the traditional topical administration of drugs. MCLs have potential beyond ocular sensing and drug delivery, and the ability of MCLs to direct and improve tear flow has demonstrated their use in alleviating dry eye problems.

In this review, we aim to present a detailed account of the developments in the field of microfluidic elements incorporated into contact lenses. In the first half of the review, we discuss the different approaches adopted for fabricating MCLs, ranging from photolithography to laser patterning. Fabrication approaches are followed by various applications of MCLs, including ocular health monitoring and drug delivery. Finally, we conclude with a brief discussion of the future prospects of microfluidic smart contact lenses.

## Fabrication approaches

### Contact lens fabrication

**Fig. 1:**
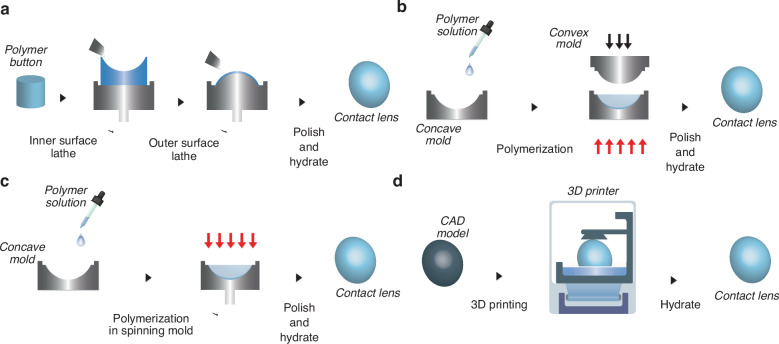
Contact lens fabrication techniques. **a** Lathe machining, **b** compression molding, **c** spin casting, and **d** 3D printing

Contact lenses are fabricated by shaping the material into prescribed curvatures, both the anterior curve and the posterior curve. The two conventional approaches to contact lens manufacturing are lathe cutting and molding. In the former approach, the lenses are carved out from the bulk material and polished, followed by hydrating, as shown in Fig. 1a. In contrast, in the latter process, a monomer solution is polymerized into the prescribed curvature using a mold. Compression molding and spin casting are two different molding approaches (Fig. 1b and c). The compression molding technique allows the anterior and posterior surfaces of the contact lens to be characterized independently by the concave and convex parts of the mold by polymerizing the liquid monomer inside a cavity matching the prescribed contact lens shapes formed by a pair of convex and concave molds. In the spin-casting method, the liquid monomer solution is spun inside a concave mold and polymerized to create thin lenses. Recent developments in material science and engineering have led to revolutionizing the manufacturing industry through the introduction of additive manufacturing or 3D printing techniques. 3D printing offers easy customization of contact lenses since the fabrication process begins with a computer-aided design (CAD) that is later converted to a machine-readable file and printed with techniques such as stereolithographic apparatus (SLA) and digital light printing (DLP), etc. (Fig. 1d)^37–39^ 3D-printed contact lenses are gaining attention owing to their ability to integrate with optical sensors and multimaterials^40,41^. Different manufacturing techniques can have a significant effect on the finished lens surface. Fine concentric rings are often observed on the surface of soft lenses manufactured through lathe cutting, whereas 3D printed lenses typically exhibit layer lines due to their layer-by-layer fabrication process. Lathe cutting and mold casting are highly scalable techniques for producing high-quality lenses, whereas modern methods such as 3D printing offer greater design flexibility and customizability. Despite its advantages, direct 3D printing of microfluidic elements onto contact lenses has not yet been reported. Therefore, the first step of integrating microfluidic elements into the contact lens is the modification of conventional fabrication approaches; some of these approaches are briefly discussed below.

### Fabrication of microfluidic contact lenses

**Fig. 2:**
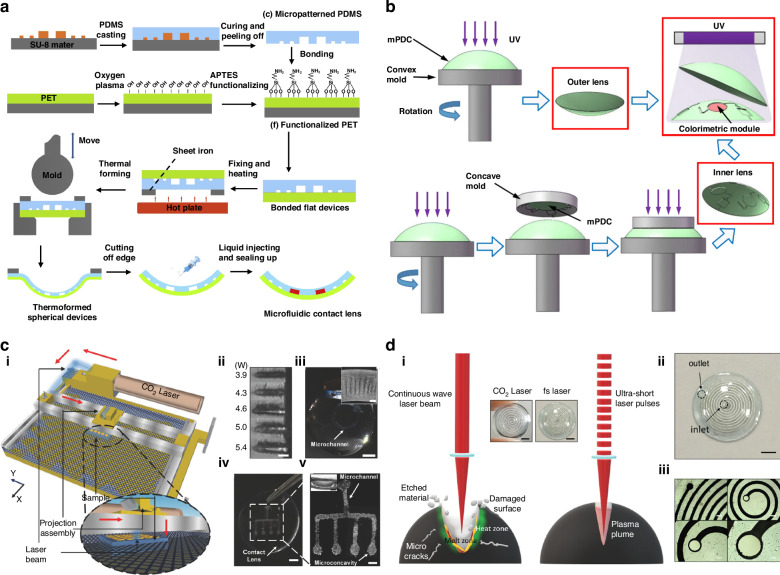
MCL fabrication techniques. **a** Schematic illustration of MCL fabrication via irreversible bonding and thermoforming, Reproduced with permission from Elsevier (2019)^45^. **b** Fabrication process of the MCL via a 3D printed mold, Reproduced with permission from Springer Nature (2020)^50^. **c** (i) Schematic representation of the CO_2_ laser patterning setup (arrows show the laser path), (ii) top view of laser patterned microcavities (scale bar = 300 µm), (iii) photographs of a laser patterned circular microchannel in the contact lens (scale bar = 300 µm), (iv) photograph of a microfluidic contact lens array via laser patterning (scale bar = 200 µm) and (v) microscopy image of a laser patterned microfluidic array in the contact lens (scale bar = 1 mm), Reproduced with permission from Wiley (2018)^54^, and **d** (i) Schematic comparison of laser ablation via a long pulse laser beam and ultrashort laser pulses, where the insets show a contact lens engraved with each process, (ii) photographs of the spiral channel engraved on a contact lens (scale bar = 5 mm), and (iii) microscopy image of the spiral channel (scale bar = 150 µm), Reproduced with permission from Wiley (2021)^57^

The fabrication or integration of microfluidic elements into contact lenses is the first and foremost step in the development of a functional MCL. Two popular approaches exist for fabricating microfluidic elements into contact lenses. The first approach involves the incorporation of microfluidic features into conventional molds and the replication of the same features onto contact lenses. The second is to embed the desired microfluidic features onto the fabricated contact lenses where the designs will be made in such a way that the microfluidic channels are devoid of the central optic zone so that the vision of the user is not affected by the fabricated structures. The first approach is essentially the soft lithography technique, which is a popular and valuable tool for integrated microfluidic system fabrication. Soft lithography includes a family of techniques involving a soft polymeric mold such as polydimethylsiloxane (PDMS) replica from an original hard master, which is typically fabricated via photolithography^42^. Since the conventional microfabrication process to obtain microfluidics devices is a planar process, employing it directly to fabricate 3D spherical devices such as contact lenses is difficult. In 2011, John Yan reported the fabrication of an MCL for IOP monitoring, using thermoforming techniques to create microfluidic elements on curved surfaces^43^. The microchannel replicated to PDMS was sealed and thermoformed (300 °C for 5 min) to create a microchannel-embedded contact lens. This method allows the fabrication of a microchannel with a width as small as 20 μm. In 2018, Hongbin et al. detailed a method of manufacturing MCLs by using irreversible bonding and thermoforming (Fig. 2a)^44^. The fabrication process involved two sets of polymers, one soft polymer (PDMS), which was used to replicate the microfeatures from the photolithographically fabricated SU-8 mold. The replicated PDMS was then chemically bonded with a polyethylene terephthalate (PET) sheet. The assembly of elastic PDMS and thermoplastic PET was thermoformed into spheres under the stamping action of a die (170 °C for 35 s). The method was able to create features of size ~40 μm and successfully created microchannels distributed linearly and annularly, which were later utilized for continuous IOP monitoring^45^. Similar to the thermoforming method, a report by Wentuo et al. uses a folding mechanism to create MCLs^46^. The fabrication method here involved the replication of a hard SU-8 master mold using PDMS followed by bonding with a thin PDMS layer. The assembly formed a notched ring shape that folded onto a sphere resembling corneal curvature, allowing 2D to 3D transformation. The empty regions in the folded microchannel assembly were filled with PDMS and cured to form microchannel-embedded contact lenses. The 2D to 3D transformation was achieved by attaching the bonded assembly of microfluidics elements onto the curved surface^47^. The 2D to 3D transformation by thermoforming or folding was time-consuming and included a complex procedure, making the scalability of the process difficult. A more direct and scalable approach was to utilize the soft lithography technique on the microfeatures embedded in the curved master mold. The incorporation of the desired microfluidic features into curved molds can significantly improve the efficiency of the fabrication approach. Researchers have used different approaches to integrate the desired microfluidics features into a curved mold. In 2020, Angelica et al. reported a method in which a metallic concave mold was engraved with microfeatures via a high-speed micromilling machine^48^. The study reported the fabrication of a 100 µm microchannel forming a double spiral design, which was later used for IOP monitoring. 3D printing is a manufacturing tool that enables the fabrication of geometrically complex devices with high precision and customization. The potential of 3D printing in the field of microfluidics has been explored by researchers, from the direct printing of microfluidic devices to the fabrication of master molds^49^. Although different 3D-printed microfluidic devices are available, there have been no reports of 3D-printed functional MCLs. Different studies have utilized the 3D printing technique to fabricate microfeatures embedded in molds and use the same for replication. This method combines the customization offered by the 3D printing technique and the scalability of conventional mold casting. The first report on the use of a 3D-printed mold for the fabrication of an MCL was from Xing et al. in 2020. The group reported the fabrication of a microfluidic network to house the sensing element and to direct tear flow for ocular sensing applications^50^. The microfluidic network included three identical sensing units for different components that were made of methacrylate poly(dodecanediol citrate), replicating a DLP 3D printed curved mold (Fig. 2b). The fabricated lenses have a very high thickness of 650 µm since the 3D-printed features itself were 350 µm in size, thus making the MCL thicker than the commercially available contact lens. The improved resolution of 3D printing techniques such as stereolithography has allowed the fabrication of smaller and more complex features on contact lenses. Recent research has reported the fabrication of microfeatures as small as 100 µm in size via stereolithography 3D printing. This method allows the fabrication of microfluidic features on hydrogels, as well as PDMS-based contact lenses^51–53^. Advancements in 3D printing techniques have great potential in improving the fabrication of MCLs, with the ultimate aim of directly printing functional MCLs.

An alternative to the replication and bonding approach for microfluidic elements fabrication is laser patterning. Surface engineering and micromachining via lasers is a well-explored area in the field of material processing. Although the concept of direct laser writing for the fabrication of microfluidic chips is widespread, in 2018, Yetisen et al. introduced it to fabricate MCLs through the integration of laser patterning and embedded templating^54,55^. A continuous wave (CW) CO_2_ laser beam mounted on a motorized translation stage was used to ablate the contact lens material to form microfluidic elements on the lens (Fig. 2c). The heat transferred due to the laser irradiation was absorbed in the hydrogel matrix to remove the polymer once the temperature reached above the decomposition temperature (360 °C)^56^. Laser-induced ablation results in the formation of a microconcavity, and when coupled with the motorized stage, researchers have realized different microfluidic networks. The laser power, exposure time, and speed of translation were varied to obtain patterns with various widths and depths, ranging from a few micrometers to hundreds of micrometers. Laser patterning was the first step involved in the fabrication of MCLs, followed by the second step of sealing the open channels with another contact lens. The laser-patterned rear contact lens is bonded with another contact lens by UV polymerization, leaving inlets and outlets for tear fluid flow. The laser patterning method offers a rapid route to create open microfluidics elements on contact lenses. However, the CW and long-pulsed lasers did not yield sharp and accurate features because of thermal effects. The availability of ultrashort lasers such as femtosecond lasers now offers an opportunity to accurately micropattern on contact lenses with minimum thermal effects, as reported by Moreddu et al. in 2021. Femtosecond laser patterning on contact lenses for fabricating different microfluidic elements, such as sensing cavities, reservoirs, and multi-inlet geometries, on fluorosilicone acrylate commercial contact lenses has been reported with resolutions of up to 80 μm (Fig. 2d)^57^. The femtosecond laser patterns were found to be more uniform and sharper than the CW CO_2_ laser patterns, and the surface topology study corroborated these findings. This approach provides the advantage of fabricating more complex networks of microfluidic elements on the contact lens. To seal the exposed microfluidic elements of the engraved acrylate, contact lenses were bonded with a PDMS-based contact lens with oxygen plasma bonding, and holes were subsequently punched for inlets and outlets. The materials used to fabricate MCLs are often not widely available in commercial contact lens materials, such as the hydrophilic core polymer poly(2-hydroxyethyl methacrylate). These hydrophilic porous, water-absorbing polymers are not suitable for microfluidic platforms because many of the constituents of the microfluidic network can diffuse over the contact lens instead of following a predictable flow pattern within the network. In this context, hydrophobic non-swelling materials such as PDMS and rigid contact lenses are more suitable options. Table 1 presents an overview of different fabrication approaches for microfluidic elements in contact lenses, highlighting their advantages and disadvantages. The comparison specifically focuses on their application in the fabrication of functional MCLs rather than a generalized assessment of these techniques. To summarize the fabrication approaches, although laser patterning offers fast and precise modifications, mold casting methods are a more scalable approach for large-scale production of MCLs. Fabrication of microfluidic features on curved molds via 3D printing techniques reduces the complexity of the otherwise complex 2D to 3D transformation by thermoforming and folding.

**Table 1:** Comparison of different techniques adopted for MCL fabrication

Fabrication technique	Advantages	Disadvantages	Applications	Maximum resolution (μm)	Process complexity	Process cost	Difficulty of batch production	References
Photolithography	• High precision and resolution	• 2D to 3D transformation of planar features is mandatory. • Process is less scalable and time-consuming.	Tear constituents, IOP monitoring, Drug delivery	15	Moderate	Moderate initial capital expenditure	High (*multistep process*)	^45–47,65,85,87^
Micro-milling	• Direct engraving on 3D molds	• Produces rougher surfaces than lithography, requiring post-processing.	IOP monitoring	100	Low	Moderate initial capital expenditure	Low (*with multiple patterned molds*)	^48,66^
Laser patterning	• A rapid and single-step process	• Requires costly tools like femtosecond lasers for precise micropatterning. • Each lens is patterned individually in a serial process.	Tear constituents monitoring	80	Moderate	High initial capital expenditure	High (*each lens is patterned individually*)	^54,56,57^
3D printing	• Good design flexibility and customizability	• Surface imperfections, such as layer lines, can compromise optical quality. • Direct printing of functional MCL remains unaccomplished.	Tear constituent monitoring, self-moisturizing, drug delivery	60	Low	Moderate initial capital expenditure	Low (*with multiple 3D-printed molds*)	^50,52,53^

## Applications

Different microfluidic elements fabricated on contact lenses are used for different applications. The broad classification of applications of MCLs is as follows: first, MCLs can be used as sensing platforms, and second, MCLs can be used as drug delivery devices. MCLs are used for sensing various ocular parameters, including different biomarkers in tear fluid or the characteristics of the eye, such as IOP and ocular surface temperature. Since MCLs are extensively used as IOP monitoring devices, this review covers them as a separate section followed by the sensing of other ocular parameters. The application section of the review concludes with a discussion on the application of MCLs in ocular drug delivery and a few other applications.

### Sensing platform

#### IOP monitoring

Glaucoma is the leading cause of irreversible blindness worldwide and is expected to affect approximately one hundred million people in the next two decades^58^. Glaucoma often presents with fluctuations in IOP; however, monitoring IOP frequently with conventional clinical measurement tools such as the Goldmann applanation tonometer is not practically feasible. Therefore, a contact lens-based minimally invasive sensor is a promising alternative. Contact lens-based IOP sensors measure changes in corneal curvature and correlate these changes to changes in IOP. One of the popular measurement techniques is electrical sensors, where the sensing elements can be capacitive, piezoresistive, etc., and where the change in corneal curvature is translated to electrical signals for IOP monitoring^15,59–61^. Over the years, other techniques for pressure monitoring have emerged, utilizing various measurement tools such as structurally colored contact lenses fabricated with submicron particle structures and pattern recognition, etc.^21,62–64^. Here, we focus on microfluidic IOP sensors, which work on the principle of microchannel deformation on contact lenses under fluctuating IOP; the deformation displaces the indicator fluid inside the channel, which is translated into IOP measurement. This innovative approach allows for continuous and noninvasive monitoring of IOP. The unique advantages of microfluidic sensors include no power requirements, biocompatibility, low cost, no electromagnetic radiation, and convenient readout through imaging.

**Fig. 3:**
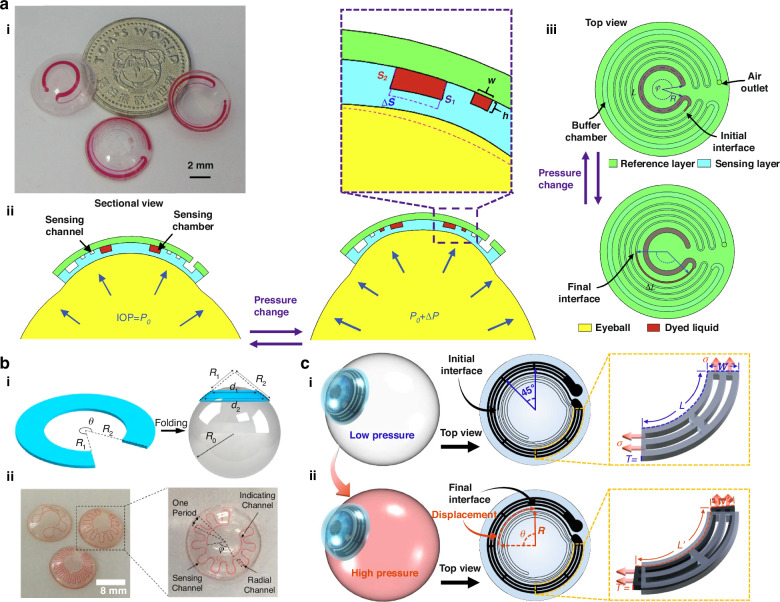
IOP monitoring using MCLs. **a** (i) Fabricated MCL for IOP monitoring, (ii) sectional view, and (iii) top view of microfluidic contact lenses under different IOPs of *P*_0_ and *P*_0_ + Δ*P*, Reproduced with permission from Elsevier (2019)^45^. **b** (i) Schematic of folding of the 2D planar notched ring into a 3D spherical ring, (ii) photograph of the MCLs of various radial microchannel periods, Reproduced with permission from AIP Publishing (2021)^46^, and **c** The operation principle of a wide bilateral wall sensing reservoir of MCL at (i) low and (ii) high IOP, Reproduced with permission from Elsevier (2021)^47^

The first report on the utilization of microchannel deformation as a yardstick for IOP measurement was by John Yan in 2011, who suggested that changes in IOP apply stress on the sensing chamber, displacing the fluid to the sensing channel^43^. The sensing platform comprises a large circular sensing chamber network followed by sensing channels, with the height of the sensing chamber being 200 μm, resulting in a bulky contact lens. The study reported that the sensing channel width has a direct correlation with the sensitivity; reducing the sensing chamber width increases the sensitivity of the device but at the expense of the dynamic range. Several different microfluidic networks have been reported for the continuous monitoring of IOP; for example, a circular microchannel placed along the periphery of a contact lens was reported by Agaoglu et al. for the measurement of IOP^65^. The volume change due to the elongation of the circular microchannel resulting from the change in IOP caused the displacement of immersion oil-filled channels to reach 108 μm per mmHg. Low sensitivity makes it challenging for the IOP variation to be read by the naked eye or smartphone. An et al. used a PET-PDMS assembly to fabricate an IOP sensor; here, the soft elastomer PDMS containing a microchannel network was bonded to a rigid PET surface. Although the configuration provided the highest reported sensitivity of 708 µm/mmHg, the process sacrificed the flexibility of the lenses (Fig. 3a)^45^. The study also indicated that a more spiral structure could enhance the sensitivity, which again was confirmed by the reports by Campigotto A. and Lai Y.^48^ Researchers designed MCLs consisting of a 100 μm wide double spiral structure and avocado oil as the indicator liquid to measure IOP. The sensitivity of the sensor decreased to 40.8 µm/mmHg, indicating that the increased surface area of the sensing chambers could provide better sensitivity. However, a similar study reported by Campigotto et al. with a new longer microfluidic configuration reported a lower sensitivity of 27.9 µm/mmHg^66,67^. Although the new study utilized a longer microchannel with a circular serpentine shape, the reduction in sensitivity can be attributed to the indicator liquid filling a very small portion of the channels. A different configuration of serpentine sensing channels 100 µm wide and deep was reported by Zhang et al. (Fig. 3b)^46^. Since the measurements were in terms of the change in central angle between the two air‒liquid interfaces on the indicating channel, a direct comparison of sensitivity was not possible. However, the volume change in the sensing channel per mmHg is 30% and 76% greater than that in previous reports^65,48^, respectively. The increased number of serpentine loops increased the sensitivity of the device, supporting the findings of previous studies. A recent report by Yuan et al. used MCL for IOP monitoring, where the microfluidic configuration consists of the usual sensing reservoir, display microchannel, and buffer chamber with the addition of a bilateral wall to improve the sensitivity^47^. An arc-shaped 75 µm wide bilateral wall was introduced into the sensing reservoir at an interval of 45° (Fig. 3c). The bilateral wall enhances the strength of the enlarged reservoir and substantially improves the sensitivity and linearity of IOP monitoring. The theoretical model predicts an increase in sensitivity with a factor of three. The study reported the highest sensitivity of a flexible MCL for a monitoring IOP of 660 μm/mmHg. The performance of different MCLs in IOP monitoring is summarized in Table 2. There have been significant improvements in terms of the manufacturing and sensitivity of devices in MCLs for IOP monitoring, and the concept has even been extended to intraocular lenses^68^. There are a few limitations, such as that most of the studies are limited to the laboratory level, improvements in the readout systems for imaging under different conditions, imaging indicator fluid displacement against different eye colors, etc.

**Table 2:** Comparison of the performance of MCLs for IOP monitoring

Authors (year)	Schematic of microfluidic architecture *(not-to-scale)*	Structural features	Indicator liquid	Detection sensitivity	Detection range (mmHg)	Reference
Yan J. (2011)		Circular sensing chambers (200 µm) followed by serpentine display channels (80 µm)	Glycerol and dye	130 μm/mmHg	46	^43^
Agaoglu et al. (2018)		Circular channels, the 5 liquid reservoir rings (50 µm) followed by sensing channel (30 µm × 30 µm)	Immersion oil	108 μm/mmHg	37.5	^65^
Chen et al. (2019)		Annular distributed sensing reservoir at 8.5 mm with display channel (150 × 40 µm)	Deionized water and glycerol and color	708 μm/mmHg	40	^45^
Campigotto et al. (2019)		Circular serpentine microchannels followed by circular display channel (100 µm)	Avocado oil and dye	27.6 μm/mmHg	10–34	^66^
Campigotto et al. (2020)		Double spiral design of 100 µm	Glycol and food color	40.8 μm/mmHg	10–34	^48^
Zhang et al. (2021)		Notched-ring structure with circular serpentine microchannels (100 µm)	Glycol and food color	1.65 deg/mmHg	–	^46^
Liu et al. (2023)		Sensing reservoir with bilateral wall structure (75 µm) followed by display channel (60 µm)	Fish oil and color	660 μm/mmHg	10–30	^47^

#### Sensing other ocular parameters

The eye contains abundant physiological information and metabolite biomarkers, and the cornea’s surface uniquely provides a convenient, noninvasive interface to the body’s physiological conditions, making contact lenses an ideal sensing platform. While most studies focus on glucose sensing^13,69^ and monitoring intraocular pressure^15,16^, contact lens-based sensing platforms have significant potential in monitoring various biomarkers such as proteins^20^, electrolytes^19^, ocular pH^18^, and temperature^70^. The conventional methods of tear sampling involve the use of Schirmer strips or capillary tubes, where the tear is collected, followed by storage and processing for further testing. Compared with these techniques, MCLs allow real-time monitoring of tear parameters via a lens, reducing the risk of damage or contamination of the samples associated with storage and transport. Fabrication of microfluidic networks consisting of various elements, such as channels, reservoirs, inlets, and outlets, can help in directing, mixing, and manipulating tear fluid on contact lenses. These microfluidic features combined with different sensing probes create a lab-on-a-contact lens platform that can monitor various ocular parameters in real-time.

**Fig. 4:**
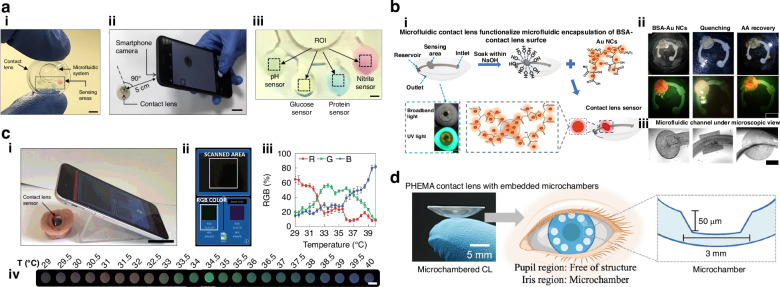
Ocular biomarker sensing using MCLs. **a** (i) The photograph of the MCL as a sensing platform (scale bar = 5 mm), (ii) the sensing arrangement using a smartphone camera, (iii) a closer look at the sensing area, Reproduced with permission from Elsevier (2020)^71^. **b** (i) The process of the implementation of the sensing elements to MCL for the ascorbic acid sensor, (ii) Photographs of encapsulated MCL and microscopic images of the channels (scale bar: 400 μm), Reproduced with permission from Elsevier (2024)^73^. **c** (i) The contact lens temperature sensor readout method, (ii) the smartphone user interface, (iii) RGB percentages, and (iv) the color palette associated with the sensor for the temperature range of 29–40 °C, Reproduced with permission from The Royal Society of Chemistry (2019)^70^. **d** Schematic illustration MCL embedded with antibody-conjugated microchambers for tear exosome detection, Reproduced with permission from Wiley (2022)^75^

*Moreddu et al*. utilized laser-patterned MCLs as a detection platform for assessing the levels of various tear components, such as glucose, nitrite, protein, and pH. The detection mechanism reported was based on the color changes that occurred as a result of the interaction between different tear constituents and the chemicals loaded in each microchamber. The microchambers were loaded with liquid colorimetric biochemical sensors selective for each tear constituent, such as hydrogen ions, glucose, protein, and nitrite (Fig. 4a). The tear fluid upon interaction with the sensors changes the color of the respective sensing chamber, which is read out via a smartphone-MATLAB algorithm interface^71^. In another study, a laser-inscribed acrylate contact lens sealed with a laboratory-made poly-HEMA contact lens was used, with a paper-based colorimetric sensing pad^72^. Similar to the previous study, the tear fluid entering the microchambers through an open inlet interacts with the paper-based sensing pads to create a color change, which is then quantified via a smartphone interface. Although the sensing elements used in both reports are the same, the paper-based sensor-incorporated contact lens showed a better limit of detection and sensitivity in most cases, possibly because of the better image contrast obtained from the paper-based sensing pads. The CO_2_ laser_-_inscribed microfluidic network was utilized by *Yetisen et al*. for multiplexed detection of physiological levels of pH, Na^+^, K^+^, Ca^2+^, Mg^2+^, and Zn^2+^ ions in tears via a microfluidic network on the scleral lens^19^. The quantification of electrolytes via fluorescence-based detection integrated with a smartphone readout system enables the differentiation of different subtypes of dry eye syndrome. In 2021*. Moreddu et al*. used a femtosecond laser to fabricate a microfluidic channel featuring an inlet, a sensing area, and a reservoir for sensing the uric acid level in tears^57^. The uricase and horseradish peroxidase-based sensor was able to detect uric acid in the range of 0–250 mg/l by coupling with reflection spectra as well as RGB color characterization. Another interesting study using paper-based sensing modules inside microcavities on contact lenses was published by *Yang et al*. ^50^ Herein, the fabrication approach adopted was different from that of the previous group, but the underlying concept was similar. The capillary flow of tears through the inlet of the microchannel connected to the sensing chamber consisting of the paper-based sensing modules allows a color change, which is then utilized for analyte quantification. Different analytes, such as chloride ions, glucose, and urea, were detected on the basis of color changes in the range of 0.078–2.5 mM. Although the fabricated lens is flexible, the increased thickness of nearly 650 μm may adversely affect user comfort. A recent work by *Shi et al*. reported the use of a laser-ablated microfluidic channel in the contact lens for the sensing of ascorbic acid, whose elevation in tear fluid is considered a marker for ocular inflammation^73^. The architecture of the microfluidic network consists of an inlet, an outlet (diameter of 0.7 and 0.9 mm), a reservoir (width of 1 mm), and a 1.73 mm diameter sensing area connected through a microfluidic channel with a width of 50 µm. The microfluidic features were engraved on a commercial soft contact lens using a UV-pulsed laser (355 nm). The sensing area was encapsulated with a bovine serum albumin-gold nanocluster, which acts as a fluorescent probe for ascorbic acid sensing (Fig. 4b). The smartphone readout system and LED excitation achieved a LOD of 0.178 mmol/L. Like tear components, the ocular surface temperature can also be used to monitor ocular physiology. Changes in ocular surface temperature are associated with different ocular conditions, such as ocular inflammation^74^. *Moreddu et al*. developed an ocular surface temperature monitoring contact lens by using microwells filled with thermochromic liquid crystal esters^70^. Their reversibility, fast response times, and high accuracy are suitable for continuous monitoring of ocular surface temperature. The temperature sensor-embedded contact lens was fabricated by creating microwells on scleral lenses via CO_2_ laser ablation and filling the well with thermochromic liquid crystals, followed by sealing with glass via UV curing. The developed lenses were able to measure temperatures in the range of 29–40 °C with a shift in the reflectance spectrum. Additionally, the smartphone-based readout system makes the whole system compact and user friendly (Fig. 4c). The latest developments in the sensing capability of contact lenses include *Li et al*. who reported the detection and quantification of exosomes. In this work, researchers illustrated the potential of antibody-conjugated microchambers for the detection of exosomes from different cell lines^75^. The microchamber design (50 µm in depth and 3 mm in diameter) was obtained via direct laser ablation on a poly 2-hydroxyethyl methacrylate (PHEMA)-based contact lens (Fig. 4d). The microchambers were fabricated on the posterior curve of the lenses, and the deep chamber provided a protective cavity for the antibodies, such that they did not rub off during contact with the cornea. The covalently attached Anti-CD81 antibodies make the device a highly promising minimally invasive monitoring platform for cancer screening. Recent developments have shown that the fabrication of microfluidic networks or microchambers is not necessary for tear fluid handling and sensing the adhesive contrast fabricated on the contact lens allows the tear film splitting and sensing of ocular biomolecules. The Tb-sensitized tear coupled with a fluorescence spectrometer allow the selective detection of lactoferrin levels in tears^76^.

### Drug delivery

Topical instillation is the most commonly used noninvasive method of administering drugs to treat eye diseases. Conventional dosage forms, such as eye drops, make up 90% of the ophthalmic formulations available on the market. This is likely due to the ease of administration and patient compliance^28,77^. The topical instillation of medications has less bioavailability and a short residence time of <2 min. This is due to anatomical and physiological factors such as tear turnover, nasolacrimal drainage, reflex blinking, and ocular static and dynamic barriers. These factors make it challenging for the medication to permeate deeper into eye tissues, with <5% of the applied dose effectively available^78,79^. To overcome the limitations of traditional ophthalmic medications, researchers are attempting to create innovative drug delivery platforms with extended retention times and high bioavailability. Drug-loaded contact lenses are now emerging as new drug-delivery tools that utilize various techniques to incorporate ophthalmic drugs into contact lenses. These lenses have garnered significant attention because of their ability to address issues related to the inaccurate administration of eye drops. Additionally, they can provide continuous drug release into the tear film, prolong the drug retention time on the ocular surface, and enhance bioavailability^28^. Although drug-loaded contact lenses have shown great potential, there are still challenges associated with them. Drug loading can alter the critical properties of contact lenses, leading to low water content, reduced oxygen and ion permeability, impaired transparency, and undesirable drug release, which may occur during postproduction monomer extraction^80,81^.

The incorporation of ophthalmologic drugs into the microfluidic components of contact lenses enables controlled and on-demand drug release. Studies have reported different drug delivery modes, ranging from the sustained release of drugs via diffusion to stimuli-responsive release. A recent study by *Kudryavtseva et al*. demonstrated a drug-eluting contact lens containing drug-loaded microchambers for the sustained release of dexamethasone, an anti-inflammatory drug^82^. In this work, researchers fabricated a donut-shaped patch containing drug-loaded microchambers that were sandwiched between hydrogel-based contact lenses. Although corticosteroid release to the eye is a diffusion-based process, the drug availability can be controlled by the size of the microchambers. However, sustained release methods can lead to suboptimal therapeutic outcomes because of the lack of flexibility in adjusting drug release rates in response to fluctuating needs. Drug release from the microreservoirs of contact lenses in response to a trigger is an ideal tactic for efficient ocular drug delivery, offering precise, on-demand treatment while minimizing drug waste.

#### MCLs with external stimuli-responsive drug release

**Fig. 5:**
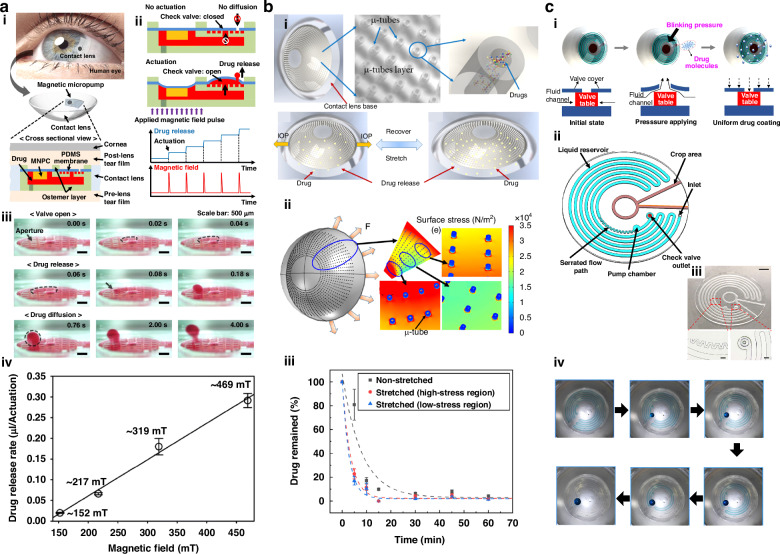
Drug delivery using MCLs. **a** (i) Schematic illustration of the magnetic micropump integrated with the contact lens, (ii) The status of the micropump and drug release under no actuation and actuation of magnetic field pulse, (iii) The sequential images showing the drug release under actuation, (iv) The quantitative evaluation of the drug release according to the strength of the magnetic field strength, Reproduced with permission from Springer Nature (2020)^83^. **b** (i) Schematic of contact lens with embedded microtubes and illustration of IOP triggered drug release, (ii) simulation results of the stress distribution on the contact lens with microtubes, iii) drug released profile from stretched devices and unstretched devices over time, Reproduced with permission from American Chemical Society (2020)^85^. **c** (i) Schematic illustration of drug release by the blinking pressure, (ii) illustration of the architecture of the microfluidic chip, and (iii) Photographs of the flat microfluidic chip (scale bar: 2 mm) and enlarged view (scale bar: 300 μm). Reproduced with permission from American Chemical Society (2022)^87^

Drug release from the microreservoirs of contact lenses in response to a trigger is an ideal tactic for efficient ocular drug delivery. In 2020, *Cong Wang* and *Jungyul Park* developed a magnetic micropump-embedded contact lens for on-demand drug delivery, and drug release was based on a magnetically actuated check valve connected to a drug-loaded chamber (Fig. 5a)^83^. A flexible layer of PDMS attached to the magnetic nanoparticle-PDMS composite opens/closes with the external magnetic field and thereby controls the release of the drug through an aperture with a diameter of 350 μm. The 500 μm-thick drug-loaded micropump was secured to the posterior surface of the contact lens, and the configuration enabled the release of the drug to the postlens tear film. The diode-like check valve on the micropump inhibited drug diffusion when no magnetic field was applied, and the release of the drug from the chamber was quick with the applied magnetic field (complete opening of the valve within 60 ms). The amount of drug displaced increased with increasing applied magnetic field strength, with a release rate of nearly 0.85 nL/mT in the applied magnetic field range of 152–469 mT. Although magnetically controllable drug release offers advantages such as a microchamber that can fill various types of drugs and operate at a magnetic field safe for human health, the thickness of the lens is nearly fivefold greater than that of commercial lenses in this case, which could lead to wearing discomfort. Additionally, the magnetic micropump requires a trigger system to provide an external magnetic field in the proximity of the contact lens. Like magnetically controllable drug release, the introduction of a flexible drug delivery system has advanced drug delivery contact lenses. The drug is loaded into the microchamber of a flexible drug delivery system with a biocompatible protection layer, which can be released from the reservoir by electrical triggering. Currently, this drug delivery system is investigated for diagnostic applications, such as continuous glucose and IOP monitoring accompanied by on-demand drug delivery. *Keum et al*. demonstrated the real-time biosensing of glucose concentrations in tears and on-demand therapeutic drug delivery from microchambers for the treatment of diabetic retinopathy in diabetic rabbit eyes^13^. In the same manner, 2022 *Kim et al*. designed a smart contact lens with a flexible drug delivery system to detect IOP and treat glaucoma^84^. These researchers developed a contact lens with a drug-loaded microchamber with a biocompatible protection layer that dissolves safely when a small, safe electric current is applied, enabling controlled drug release without harming the eye.

#### MCLs with internal stimuli-responsive drug release

Although externally triggered drug release offers more control over drug delivery, additional involvement of the user or an interface is needed. An ideal approach would be a drug delivery platform that evaluates the changes in ocular condition and responds correspondingly, or in other words, an internal stimulus. *Ding et al*. introduced a microtube-embedded contact lens for self-adaptive drug release for treating glaucoma patients (Fig. 5b)^85^. Herein, a microtube array on PDMS replicated from a photolithographically generated master was attached to a PDMS contact lens. The microtube array houses the drug and is released into the ocular environment via diffusion. The diffusion-based drug release was driven by the concentration gradient and thereby offered an opportunity to control the diffusion of the drug by changing the microtube dimensions. Although a 45 μm wide microtube with a depth of 60 μm was able to slow the diffusion from other combinations presented in the work, 80% of the drug was released within the first hour of wearing. Increasing the microtube density and tuning the tube dimensions could help increase the drug retaining capacity, but the outburst of the drug in the initial stage of wearing may result in adverse effects. The adaptive drug release of microtubes was based on the microtube shape deformation induced by the fluctuating IOP. The increased IOP stretches the microtubes to accelerate the diffusion rate. Recently, a fish fin structure inspired by microstructures on contact lenses was proposed by *Li et al*. for adaptively adjusting the drug release rate on the basis of changes in IOP^86^. Liquid drugs are stored within the interstices of deformable microstructural units, and changes in IOP achieve adaptive drug release. In vivo animal studies have also shown that high IOP in rabbits effectively trigger drug release and reduces IOP, indicating its favorable therapeutic efficacy. IOP-induced drug delivery is an ideal technique for drug release in which physiological changes trigger drug release, which will restore the system to a normal state. Another example of the use of an internal trigger for drug release from microfluidic element-embedded contact lenses was demonstrated by *Du et al*. in 2022, where the drug was released from the microchamber by exploiting the blinking pressure (Fig. 5c)^87^. In this research, concentric arc-shaped channels with widths and heights of 300 and 80 μm, respectively, were connected to a pressure-triggered valve with a diameter of 210 μm. The blinking induced a rise in pressure, which opened the valve in the closing phase of the blink, triggering the release of the drug and restoring its original state when the eyelid pressure was removed by the opening phase of the blink. The arc-shaped channels can house nearly 3.5 μl of drugs and offer flexibility in selecting drugs. The threshold pressure required to open the check valve was set to 7.2 kPa, which was lower than the average eyelid pressure of 9 kPa. The pressure required to open the valve was optimized by controlling the thickness of the PDMS layer; a thicker layer led to an increased pressure requirement, and the optimized pressure corresponded to a 34 μm-thick layer. Although blinking induces drug release and can provide the means for the continuous and controlled release of drugs into the ocular environment, the inconsistencies in eyelid pressure and blinking abnormalities associated with different ocular complications pose challenges to the proposed strategy. Recent advances in drug delivery MCLs include the pH-responsive drug release demonstrated by *Aravind et al*. The drug-loaded hydrogel is incorporated in the spherical cavity with a narrow opening, which restricts diffusion to a minimum under normal ocular pH conditions (pH > 6). When the ocular pH decreases (pH < 6), the pH-responsive hydrogel inside the spherical cavity de-swells to accelerate the drug release^88^. To summarize, compared with diffusion-based drug-eluting contact lenses, MCLs offer controlled and on-demand drug release with external and internal release triggers. However, further development of fabrication approaches is needed for the facile and scalable production of MCLs for ocular drug delivery.

### Other applications

**Fig. 6:**
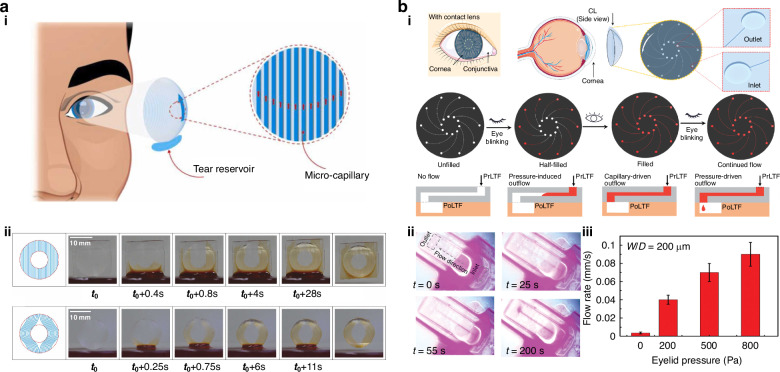
MCLs for managing contact lens induced dry eye. **a** The illustration of the working of self-moisturizing contact lens employing capillary flow. **b** The sequential images showing capillary action on microchannel embedded contact lens, Reproduced with permission from Elsevier (2022)^52^. (i) Schematic of the eyelid pressure enhanced tear exchange through microfluidic channels. (ii) The sequential images showing pressure-induced outflow in hydrated hydrogel microchannels. (iii) The effect of eyelid pressure on flow rate within hydrated microchannels, Reproduced with permission from Wiley (2022)^53^

The applications of MCLs are not limited to ocular health monitoring and drug delivery. MCLs have the potential to manipulate the flow of tear film and improve ocular health. Dry eye disease is one of the major reasons for contact lens discontinuation, and contact lens-induced dry eye (CLIDE) can lead to other ocular complications, including infections^89^. Researchers have been attempting to increase the availability of tears on the contact lens surface to reduce the incidence of complications associated with CLIDE. Improving the wettability of contact lenses via surface modifications or the addition of wetting agents can increase the integrity of the tear film^90^. The incorporation of graphene on contact lenses reduces evaporation through the lenses, as demonstrated by *Lee et al*. ^91^ However, recently, a new class of contact lenses, called self-moisturizing contact lenses, was developed in which the contact lens surface is kept wet by driving tears to the lens surface via different techniques. *Kusuma et al*. developed self-moisturizing contact lenses, where the tear film was driven through the contact lens surface by electroosmotic flow with the help of a biocompatible battery-integrated contact lens^92^. Although osmotic flow can successfully keep the lens surface moist, the process requires an external power supply. Later, in 2022, *Aravind et al*. demonstrated the fabrication and self-moisturizing action of microfluidic channel-embedded contact lenses. The work employed a capillary flow of liquid through an array of open microfluidic channels decorated on the contact lens surface to keep the surface moist (Fig. 6a)^52^. Microfluidic elements were designed for the CAD model of the mold and 3D printed, followed by a mold casting method to replicate the structures on the contact lenses. The capillary flow of the tear fluid through the array of microchannels replicated on the lens surface keeps the lens from drying. Since the employed 100-micron wide channels cause scattering and reduce the optical transparency of the lens, the central optical zone was made devoid of moisturizing microchannels. Although the capillary array allows the surface to remain moist, the exchange between the prelens tear film and the postlens tear film is a serious concern in contact lens users. Recently, *Zhu et al*. designed a contact lens where tear exchange through the contact lens was realized with microchannels (Fig. 6b)^53^. The microchannels in the bulk of poly(HEMA) with an inlet and outlet on either side of the material are fabricated with the assistance of 3D printing followed by replication. The method adopted allows the fabrication of different geometries and dimensions ranging from 30 to 400 µm. The proof of concept also demonstrated that the blinking pressure (0.1–5 kPa) enhanced tear exchange. Another important aspect in regard to dry eye disease mitigation is the quantification of tear volume. The conventional methods of tear volume analysis include Schirmer’s test and capillary tube methods. In 2021, *Rosalia Moreddu et al*. reported a tear fluid volume wearable sensor that was based purely on a microfluidics technique^57^. The spiral microfluidic channel-engraved contact lens with a femtosecond laser was used as a visual method on the basis of the wetted spiral area after 10 s of wear. This spiral microchannel has the potential to act as a tear fluid volume sensor as well as a tear fluid sampling tool.

## Conclusion and future outlook

It is well known that contact lenses are widely used for vision correction. Hence, leveraging this platform for ocular diagnosis, drug delivery, etc. is an additional benefit. In this review, we presented an overview of the fabrication of MCLs and their applications in ocular diagnostics and treatment. MCLs offer several advantages, such as the ability to process on-eye real-time tear fluid, no power requirements, biocompatibility, and low cost. The unique advantage of MCLs is the efficient utilization of the limited amount of tear fluid available in the minimum space accessible on a contact lens. The first and foremost step in the realization of a functional MCL is the fabrication of micron-scale structural features on the contact lens. Although laser-assisted fabrication approaches have shown great promise, the scalable fabrication of MCLs heavily depends on the 2D to 3D conversion of soft-lithographically replicated planar structures. Nevertheless, advances in 3D printing techniques for the fabrication of microfluidic feature-integrated curved molds are expected to provide an impetus for the fabrication of commercial MCLs for diverse applications. MCLs use microstructures to store and direct tear fluid and allow efficient manipulation of a limited volume of tears via microfluidic networks. In addition, such microfluidic channels integrated with different sensing elements on MCLs could provide improved sensing accuracy and reliability. Wearable sensing platforms such as MCL present an efficient and patient-centric solution that addresses the challenges of conventional tear fluid analysis methods. They enable continuous, noninvasive, and real-time monitoring of tear fluid composition by eliminating the need for sample collection and processing. Additionally, after the initial device setup, these platforms could significantly reduce the recurring costs associated with laboratory tests. When MCLs are integrated with different sensing techniques, such as colorimetry and spectroscopy, they have great potential for the sensitive and selective detection and quantification of various ocular markers, such as proteins, electrolytes, pH, and temperature, etc, and can be extended for routine clinical applications. Continuous IOP monitoring is critical for assessing ocular health and preventing glaucoma. Traditionally, IOP is measured via tonometry performed by ophthalmologists. However, this approach has significant limitations, as it requires patients to visit a healthcare facility, restricting measurements to specific times. Additionally, IOP levels are influenced by factors such as body posture and diurnal variations, with fluctuations differing between healthy individuals and glaucoma patients. To address these challenges, continuous IOP monitoring is essential, and MCLs offer a convenient, self-operable, and efficient solution. Unpowered continuous IOP monitoring using MCLs has emerged as a highly sensitive and innovative tool for glaucoma management. The general principle behind real-time IOP monitoring with MCLs involves a flexible microchannel network filled with indicator fluid embedded within the lenses. This system measures IOP fluctuations by detecting fluid displacement within the microchannels, providing a direct and accurate assessment of IOP changes over time. Most IOP monitoring and several colorimetric sensor-integrated MCLs depend on an imaging-based smartphone interface for the interpretation of the data. The readout system, which is based on mobile applications, allows real-time data logging and transfer to clinicians for efficient diagnosis. MCLs for drug delivery are an emerging area, and microfluidic elements such as cavities and channels allow the housing of different ocular medications and their release under external or internal triggers. The on-demand supply of medication in response to the associated changes in ocular parameters is the ideal strategy, and MCLs can achieve this goal without compromising structural integrity or permeability. The integration of microfluidics into contact lenses has applications beyond ocular sensing and drug delivery, and the ability of MCLs to direct and improve tear flow has demonstrated their potential for alleviating CLIDE. It is crucial to overcome the clinical barriers associated with MCLs to ensure that they are comfortable, durable, and safe for extended use. Rigorous clinical trials are essential to address these challenges, validate their performance, and ensure their suitability for long-term wear in real-world applications.

Although strategies for fabricating MCLs have been developed over the past few years, the large-scale fabrication of MCLs is still a challenging task, as most of these techniques rely on the 3D transformation of conventionally fabricated planar microstructures. Although few studies have adopted 3D printing techniques and curved molds, the fabrication of closed microfeatures on lenses is still a time-consuming and labor-intensive process and requires further research. Nevertheless, we expect that in the future, direct 3D printing of MCLs will be a reliable fabrication tool, which could lead to fast and customized production of MCLs. Calorimetric detection and smartphone-based imaging readout systems are convenient approaches integrated with MCLs for ocular diagnosis. Improvements in imaging systems against different environments and eye colors will enhance the MCL’s sensing capabilities along with additional optimization of the effects of eye movements, blinking, and interpersonal eye features. In addition to these challenges, smart MCLs capable of performing multiple diagnostics and supplying essential drug loads on the basis of changes in the ocular environment constitute a significant step toward better healthcare.
